# Overall survival, adverse events, and economic burden in patients with chronic lymphocytic leukemia receiving systemic therapy: Real‐world evidence from the medicare population

**DOI:** 10.1002/cam4.3855

**Published:** 2021-03-18

**Authors:** Ravi K. Goyal, Saurabh P. Nagar, Shaum M. Kabadi, Hannah Le, Keith L. Davis, James A. Kaye

**Affiliations:** ^1^ RTI Health Solutions Research Triangle Park NC USA; ^2^ AstraZeneca Gaithersburg MD USA; ^3^ RTI Health Solutions Waltham MA USA

**Keywords:** adverse events, chronic lymphocytic leukemia, CLL, costs, overall survival, treatment patterns

## Abstract

**Background:**

Information on overall survival (OS) and adverse events (AEs) in patients with chronic lymphocytic leukemia (CLL) is mostly available from clinical trials. We therefore conducted a population‐based retrospective cohort study to assess OS, incidence of AEs, and economic burden in real‐world practice among Medicare patients treated for CLL.

**Methods:**

Patients with CLL receiving ≥1 systemic therapy from 2013 to 2015 were selected from the Medicare claims database and followed from the start of first observed systemic therapy (index date) through December 2016 or death. OS for patients receiving each of the most commonly observed treatments was estimated by the Kaplan–Meier method. AEs were assessed among patients receiving these treatments across all observed lines of therapy. All‐cause direct medical costs were assessed from the Medicare system perspective.

**Results:**

Among 7,965 eligible patients across all observed therapy lines, ibrutinib monotherapy (Ibr; *n* = 2,708), chlorambucil monotherapy (Clb; *n* = 1,620), and bendamustine/rituximab (BR; *n* = 1,485) were the most common treatments. For first observed therapy, 24‐month OS estimates for Ibr, Clb, and BR recipients were 69% (95% CI = 68%–71%), 68% (95% CI = 65%–71%), and 79% (95% CI = 77%–81%) respectively. The most frequently recorded AEs in patients receiving these treatments in any observed line of therapy were neutropenia, hypertension, anemia, and infection. For all patients, the mean monthly all‐cause cost during the follow‐up period was $8,974 (SD = $11,562); cost increased by the number of AEs, from $5,144 (SD = $5,409) among those with 1–2 AEs to $10,077 (SD = $12,542) among those with ≥6 AEs.

**Conclusion:**

Over two‐thirds of patients survived at least 2 years after starting their first observed therapy for CLL. Our findings highlight considerable susceptibility to AEs and unmet medical need in Medicare patients with CLL treated in routine practice. Medicare incurred substantial economic burden following initiation of systemic therapy, and patients with greater numbers of AEs accounted disproportionately for the high overall cost of CLL management.

## Graphical abstract

A population‐based retrospective analysis was conducted to document treatment patterns, overall survival, adverse events, and economic burden in Medicare patients with chronic lymphocytic leukemia (CLL). Findings demonstrate that over two‐thirds of patients survived at least 2 years after the start of their first observed therapy during the study period and that greater number of adverse events were associated with higher economic burden of CLL.

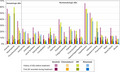

## 1 BACKGROUND

Chronic lymphocytic leukemia (CLL), the most common type of leukemia, represents more than one‐third (37%) of all newly diagnosed leukemia cases in the United States (US).^1^ It is typically a slowly progressing disease; the median age at diagnosis is reported to be between 70 and 72 years.^2^ Most patients are diagnosed at early stages and do not require treatment until symptoms develop and there is clinical evidence of disease progression.^3^

According to the National Comprehensive Cancer Network (NCCN) guidelines for CLL (2020 version), in frail patients with significant comorbidity, patients aged ≥65 years, and in younger patients with significant comorbidities, with or without del(17p)/p53 mutation, the preferred first‐line therapies include ibrutinib (a Bruton's tyrosine kinase [BTK] inhibitor), acalabrutinib (a second‐generation BTK inhibitor) alone or in combination with obinutuzumab (an anti‐CD20 monoclonal antibody), and venetoclax (a BCL2 inhibitor) in combination with obinutuzumab.^3^ The combination therapies of bendamustine (an alkylating agent with antimetabolite properties) and chlorambucil (another alkylating agent) with an anti‐CD20 monoclonal antibody, which were previously the preferred first‐line regimens, have been included as “other recommended therapies” in the current treatment guidelines.^3^ Recommendations for patients with relapsed or refractory CLL, with or without 17p deletion, currently include one of the following preferred therapies: acalabrutinib, ibrutinib, venetoclax, venetoclax/rituximab, the combination of idelalisib (a phosphoinositide 3‐kinase [PI3 K] inhibitor) with rituximab; and duvelisib (another PI3 K inhibitor).^3^

As with most cancer‐directed systemic therapies, treatments for CLL frequently lead to hematologic and nonhematologic adverse events (AEs). These AEs, if severe enough, may necessitate medical intervention and disrupt planned treatment schedule, which in turn may lead to suboptimal health outcomes and increased expenditures. The AE burden in patients with CLL has been well‐documented in clinical trials; however, confirmatory experience from real‐world data, particularly among Medicare beneficiaries, remains scarce. Recent advances in the treatment of CLL, especially the availability of targeted therapies, have led to improvement in survival, as the CLL‐related death rate steadily reduced by approximately 3% per year between 2006 and 2015.^4^ The incidence of CLL, however, has increased from 7,300 cases in 1998 to 20,940 cases in 2018, probably owing to the aging US population.^5^ The American Cancer Society estimated a total of 21,040 new cases of CLL and 4,060 deaths from CLL in the US in 2020.^6^ The annual economic burden of CLL in the US is projected to increase almost sevenfold from approximately $740 million in 2011 to more than $5 billion by 2025.^7^ A limited number of population‐based observational studies have explored treatment utilization patterns and health outcomes, including AE burden, survival, and/or costs, in patients with CLL in the US. Also, these studies have several limitations: they represent a commercially enrolled population^8^ or a small (5%) subset of the Medicare population^9^; use data from a period prior to the availability and broad uptake of newer targeted therapies^10^, ^11^, ^12^; only report data on a limited set of outcomes^13^; or report outcomes only in patients receiving one specific type of treatment.^14^ In this study, we aimed to address these gaps by providing a more comprehensive account of overall survival (OS), AEs, and economic burden among patients with CLL treated with systemic therapies in the US Medicare population. At the time of this study, Medicare data were available through 2016, and therefore, this present study includes only the findings related to treatment agents that were available during the study period.

## 2 METHODS

### 2.1 Design and data source

For this retrospective cohort study, the Medicare Research Identifiable Files (RIF) (2012–2016), which contain nation‐wide administrative health care claims data of Medicare beneficiaries, were obtained from the Centers for Medicare and Medicaid Services. The Medicare RIF data include detailed information on beneficiary enrollment, demographics, claims‐level procedures and diagnoses received, dates of visits/services, provider type, type of service, setting of care, and the charges and paid amounts. Medicare RIF data also include information on beneficiaries’ vital status and the date of death if applicable.

### 2.2 Patient selection

Patients initiating a CLL‐directed systemic treatment during the patient selection window (from July 1, 2013, through December 31, 2015) were identified. The systemic treatments of CLL were identified from the NCCN guideline recommendations for CLL (version 2, 2017) available at the time of study initiation.

The diagnosis of CLL was ascertained based on the presence of at least two claims, on separate days, with a diagnosis code for CLL (*International Classification of Diseases*, *Ninth Revision*, *Clinical Modification* [ICD‐9‐CM: 204.1x] or *International Classification of Diseases*, *Tenth Revision*, *Clinical Modification* [ICD‐10‐CM: C91.1x]) on or before the date of first observed treatment for CLL (as such diagnosis codes for CLL were observable during Jan 1, 2012, through Dec 31, 2015). Small lymphocytic lymphoma (SLL), which has biologic characteristics same as CLL but with different clinical manifestations, was considered to represent the same disease as CLL, and therefore, its diagnosis code (ICD‐10‐CM: C83.0x) was also included in the algorithm to identify patients with CLL. The date of the first observed systemic treatment for CLL during the patient selection window defined the study index date. Subsequently, patients were included if they (a) were at least 18 years of age at the index date; (b) had at least 18 months of continuous enrollment in Medicare Parts A, B, and D plans before the study index date, with no enrollment in health maintenance organization plans; and (c) did not have any evidence of CLL‐directed treatment (systemic therapy and/or stem cell transplant) in the claims‐history available before the index date (starting from Jan 1, 2012) in order to improve accuracy about the “line” of therapy observed. The limited years of study data available prior to index date for this research, however, did not allow analyses by actual lines of therapy; therefore, in this article, we report findings only within the context of “observed” lines of therapy. All patients were followed from their study index date through death, Medicare disenrollment, or the end of the study period (December 31, 2016), whichever was the earliest. Of note, because ibrutinib received approval for CLL in 2014, the follow‐up time for patients receiving this drug was expected to be shorter than that for patients receiving other treatments. A study design schematic is presented in Figure S1. Cohort selection and stepwise sample attrition are further described in the patient selection flowchart (Figure S2).

### 2.3 Study measures

#### 2.3.1 Baseline patient characteristics

Patient demographics, including age at the index date, gender, race, year of the study index date, and geographic region, were assessed. Charlson Comorbidity Index (CCI) was calculated using diagnosis codes observed during the baseline period to assess patients’ comorbidity burden at the time of index date.^15^ In addition, patients’ baseline risk of atrial fibrillation was assessed based on previously published methods,^16^ which accounted for seven risk factors, including (1) heart failure, (2) hypertension, (3) diabetes, (4) age 65 to 74 years (at the index date), (5) age ≥75 years (at the index date), (6) coronary artery disease, and (7) chronic kidney disease. Patients were classified as “high‐risk” if they met any of the following three criteria: had any two of the first five risk factors listed above, had any three of all seven risk factors listed above, or had a previous diagnostic code for atrial fibrillation (ICD‐9‐CM: 427.31, or ICD‐10‐CM: I48.x, I48.9x).

#### 2.3.2 Treatment characteristics

The CLL‐directed systemic therapies assessed in this study were based on the NCCN Clinical Practice Guidelines for CLL/SLL (version 2, 2017; published February 2017), which we expected to encompass all treatments that were available over the study period ending in December 31, 2016.^17^, ^18^ Treatments were identified using medical and pharmacy claims containing treatment‐specific procedure codes (for drugs covered under Part B plan) or prescription drug codes (for drugs covered under Part D plan). The date of the first claim with a systemic therapy agent defined the start of the first “observed line of therapy” (i.e., same as the index date). Therapy regimens and observed lines of therapy were defined following previously published methods.^19^, ^20^, ^21^, ^22^ Detailed characteristics on each therapy regimen initiated on and after the study index date, including regimen composition, time to initiation, duration of therapy, were assessed. We also defined maintenance therapy when a rituximab monotherapy was initiated in the second or later observed lines within 7 months after completion of a previous rituximab‐containing combination therapy, such as bendamustine/rituximab (BR).

#### 2.3.3 Overall survival

Overall survival was defined as time from the study index date (i.e., start of the first observed line of therapy) to death (all‐cause) or the end of follow‐up (Dec 31, 2016). Patients who were still alive (based on no observed record of death) at last available follow‐up were censored in the survival analysis. The median survival time (in months) was analyzed separately for the four most commonly observed therapy regimens. The number and proportion of patients who died during the follow‐up period were also summarized.

#### 2.3.4 Adverse events

Hematologic and nonhematologic AEs during CLL therapies were assessed for the most commonly observed regimens across all observed lines of therapy in the follow‐up period. AEs were assessed as the first observed occurrence based on the presence of at least one claim with AE‐specific diagnosis code(s) during the treatment. AEs were counted by using two approaches: one approach counted AEs regardless of any observed history of the AE before treatment initiation (“prevalent”) and the other approach counted only new AEs (i.e., no observed history of the same type of event before start of the treatment [“incident”]). The rationale for using this method to define AEs was that incident AEs may be more likely to be caused by treatment, but they probably underestimate the actual incidence of AEs, which the prevalent AEs are more likely to estimate. In addition, the total number of unique AEs incurred over the study follow‐up period (encompassing all observed therapy lines) was recorded for each patient and categorized into the following groups: 0, 1–2, 3–5, or ≥6 AEs. The number of unique AEs experienced during the first observed line of therapy was also assessed.

#### 2.3.5 Health care resource use and costs

We analyzed mean per patient per month (PPPM) health care resource utilization (HCRU) and costs during the active treatment as well as during all available follow‐up period after treatment initiation (encompassing all lines of therapy observed). Both all‐cause and CLL‐related HCRU and costs were assessed for the overall cohort and by care setting (i.e., inpatient, emergency department, hospice, office, hospital outpatient, and skilled nursing facility). CLL‐related measures were defined by using the subset of claims with (a) a diagnosis code for CLL in the primary or secondary position in medical claims file or (b) a treatment code for CLL‐directed therapies (e.g., chemotherapy) in medical and pharmacy claims files. AE‐related costs were defined by using the paid amounts associated with claims containing a diagnosis code (at primary position or elsewhere) for the AE in question during the first observed line of therapy. AE claims that occurred during the line of therapy were included irrespective of prior AE history. All cost data represented final amounts paid to providers by Medicare for services and treatments delivered.

### 2.4 Data analysis

Descriptive statistics were used to summarize study measures, including patient characteristics, treatment patterns, AEs, and survival outcomes. Median OS and 95% confidence intervals (CIs) were estimated by using the Kaplan–Meier method separately for the most common therapy regimens observed. The 24‐month OS rates and 95% CIs were also analyzed. The HCRU and direct medical costs in the follow‐up period were assessed overall and by care setting, and the mean PPPM estimates, which standardized the data by accounting for variability in length of follow‐up, were reported. All cost data were adjusted to 2017 US dollars by using the medical care component of the US Consumer Price Index since this was the most recent year for which the annual inflation factor was available at the start of study analyses. Costs were assessed from the payer's perspective and included health plan paid amounts and the coordination of benefit amounts. To explore incremental costs associated with AEs, we stratified the mean PPPM costs by the total number of AEs experienced during the follow‐up period (i.e., 0, 1–2, 3–5, and ≥6 AEs).

We also performed multivariable logistic regression analysis to assess factors associated with inpatient admission and multivariable generalized linear model to assess factors associated with monthly costs. Both models assessed correlations with (not causation of) the outcomes during the first observed line of therapy. A categorical variable representing the total number of unique AEs experienced during the first observed line of therapy (0–2 and ≥3 AEs) was included in these models as the primary independent variable to assess incremental inpatient admission and cost burdens associated with more AEs. All analyses were performed by using SAS statistical software, version 9.4 (SAS Institute Inc.; 2011).

## 3 RESULTS

### 3.1 Baseline patient characteristics

A total of 7,965 patients met the study inclusion criteria (median age, 76 years [range, 32–104 years]; 56% male and 91% white). Patients had a high comorbidity burden, with a mean CCI score of 4.7 (standard deviation [SD] = 3.4) in the 18‐month baseline period and a mean daily pill burden of 4.1 (SD = 3.4) in the month before the study index date. More than three‐fourths (82%) were at “high” risk of A‐fib at baseline. The mean of monthly all‐cause costs over the baseline period was $1,865 (SD = $2,347). A detailed description of baseline patient characteristics, by treatment group, is provided in Table 1.

**TABLE 1:** Baseline characteristics of patients with CLL assessed during the preindex period

	Ibrutinib		Chlorambucil		BR		Rituximab	
All patients, N (%)	2,033	100.0%	1,539	100.0%	1,310	100.0%	1,063	13.3%
Age at index, y								
Mean (SD)	75.2	8.4	79.6	8.2	74.5	7.4	78.4	8.2
Median	75		80		74		79	
Min, Max	41	101	44	102	32	96	46	104
Male, n (%)	1,231	60.6%	757	49.2%	810	61.8%	543	51.1%
Race, n (%)								
White	1,787	87.9%	1,370	89.0%	1,194	91.2%	998	93.9%
Black	179	8.8%	125	8.1%	77	5.9%	39	3.7%
Others/unknown	67	3.3%	44	2.9%	39	3.0%	26	2.5%
Year of study index date (treatment), n (%)								
2013	31	1.5%	281	18.3%	336	25.6%	225	21.2%
2014	985	48.5%	675	43.9%	511	39.0%	437	41.1%
2015	1,017	50.0%	583	37.9%	463	35.3%	401	37.7%
Length of follow‐up, d^a^								
Mean (SD)	578.8	289.5	632	326.9	719.9	339.6	679.2	329.5
Median	591		630		725		684	
Min, max	3	1135	3	1277	1	1279	2	1270
Atrial fibrillation risk status, n (%)^b^								
High‐risk	1,578	77.6%	1,346	87.5%	1,034	78.9%	908	85.4%
Low‐risk	455	22.4%	193	12.5%	276	21.1%	155	14.6%
CCI score								
Mean (SD)	4.6	3.3	5	3.6	4.2	3.1	5.1	3.4
Median	4		4		4		5	
Min, max	0	17	0	22	0	17	0	20
Average monthly costs^c^								
Mean (SD)	$2,726	$2,991	$1,609	$2,063	$1,257	$1,491	$1,786	$2,391
Median	$1,920		$937		$801		$1,050	
Min, max	$54	$46,788	$16	$25,267	$60	$25,393	$46	$36,387
Death during study	617	30.30%	522	33.90%	288	22.00%	304	28.60%

Abbreviations: BR, bendamustine +rituximab; CCI, Charlson Comorbidity Index; CLL, chronic lymphocytic leukemia; SD, standard deviation.

^a^ Follow‐up time calculated as number of days between the study index date and the end of the follow‐up or death.

^b^ Patients were defined as “high risk” for atrial fibrillation if there was evidence of ≥2 of the first 5 or ≥3 of the 7 risk factors (heart failure, hypertension, diabetes, age 65–74 years, age ≥75 years, coronary artery disease, chronic kidney disease) present in the 12‐month baseline period using the method used by Chyou and colleagues,^16^ or if patient had prior history of atrial fibrillation during the baseline period.

^c^ Mean monthly all‐cause costs over the 12‐month baseline period (includes costs for inpatient stays, emergency department visits, office visits, other outpatient and ancillary care, and pharmacy visits).

### 3.2 Treatment characteristics

All patients (*n* = 7,965) received a first observed systemic therapy line in accordance with the eligibility requirements; 1,940 (24.4%) received a second, 450 (5.6%) received a third, and 124 (1.6%) received a fourth observed systemic therapy line during the study period (Table 2). For first observed therapy line, ibrutinib monotherapy (*n* = 2,033 [25.5%]) was the most frequent treatment regimen followed by chlorambucil monotherapy (*n* = 1,539 [19.3%]), BR (*n* = 1,310 [16.5%]), and rituximab monotherapy (*n* = 1,063 [13.3%]); the remaining 24.4% received other treatments (Table 2). We focused on analyzing and reporting data for the four most frequently used treatment regimens as observed in the data because these regimen groups had adequate sample sizes to allow a meaningful analysis of the study outcomes by treatment type.

**TABLE 2:** Distribution of systemic therapy regimens by observed line

First Observed Line (N = 7,965)			Second Observed Line (N = 1,940)			Third Observed Line (N = 450)			Fourth Observed Line (N = 124)		
	N	%		N	%		N	%		N	%
Ibrutinib	2,033	26	Ibrutinib	678	35	Ibrutinib	140	31	Rituximab	43	35
Chlorambucil	1,539	19	Rituximab	298	15	Rituximab	95	21	Ibrutinib	31	25
BR	1,310	16	Chlorambucil	206	11	Chlorambucil	41	9	Other regimens	50	40
Rituximab	1,063	13	BR	175	9	BR	23	5			
Unclassified biologics	232	3	Obinutuzumab	59	3	Unclassified biologics	14	3			
Obinutuzumab‐chlorambucil	205	3	Obinutuzumab‐chlorambucil	52	3	Obinutuzumab‐chlorambucil	12	3			
Methotrexate	171	2	Bendamustine	39	2	Other regimens	125	28			
FCR	157	2	Ibrutinib‐rituximab	38	2						
Bendamustine	149	2	Unclassified biologics	33	2						
Carboplatin	146	2	Methotrexate	31	2						
Other regimens	960	12	Other regimens	331	17						

Note

In accordance with a requirement of the Medicare data use agreement, data on categories with cell sizes 1 through 10 must be suppressed; therefore, in the third and fourth observed lines, several regimens with low frequencies are grouped within the “Other regimens” category.

Abbreviations: BR, bendamustine +rituximab; FCR, fludarabine +rituximab + cyclophosphamide; RCHOP, rituximab +cyclophosphamide + doxorubicin +vincristine ± prednisone.

The median duration of exposure for the most common first observed treatment regimens was as follows: ibrutinib monotherapy, 10.4 months (quartile 1 [Q1] = 6.0, quartile 3 [Q3] = 17.6); chlorambucil, 3.0 months (Q1 = 1.2, Q3 = 5.6); BR, 4.7 months (Q1 = 2.9, Q3 = 5.8); and rituximab monotherapy, 3.6 months (Q1 = 1.7, Q3 = 2.8). Among the first observed treatments, 16.7% of patients receiving ibrutinib were still on therapy at the end of study follow‐up. Among patients who received ibrutinib in the first observed line and discontinued (for reasons other than the end of study follow‐up or death), 6.3% reinitiated treatment with ibrutinib in the second observed line. Rituximab maintenance therapy utilization was 14.8% following the first observed therapy line (median duration = 7.2 months), 11.3% following the second observed therapy line (median duration = 1.7 months), and 10.5% following the third observed therapy line (median duration = 1.7 months). The median length of follow‐up from the index date through the end of study period was 19 months for patients whose first observed therapy was ibrutinib, 21 months for those for whom it was chlorambucil, 24 months for those who received BR, and 22 months for the rituximab group.

Among the newer targeted therapies (other than ibrutinib), utilization across all observed therapy lines ranged from <1% to 2.4% of patients for ofatumumab (alone or in combination with chemotherapy), from 3.2% to 6.4% for obinutuzumab (alone or in combination with chemotherapy), and from 1.4% to 3.1% for idelalisib. Venetoclax was used in less than 1% of patients across all observed lines.

### 3.3 Overall survival

With median follow‐up less than 24 months for all treatment cohorts, the estimated median OS was 40.8 months (95% CI, 38.6‐not reached) in the chlorambucil group and was not yet reached in the other three treatment groups (Figure S3). The 24‐month OS estimates were 69% (95% CI, 67.6%–71%) in the ibrutinib group, 79% (95% CI, 77.4%–81.1%) in the BR group, 68% (95% CI, 64.9%–71%) in the chlorambucil group, and 74% (95% CI, 70.9%–76.5%) in the rituximab group.

### 3.4 Adverse events

**FIGURE 1:**
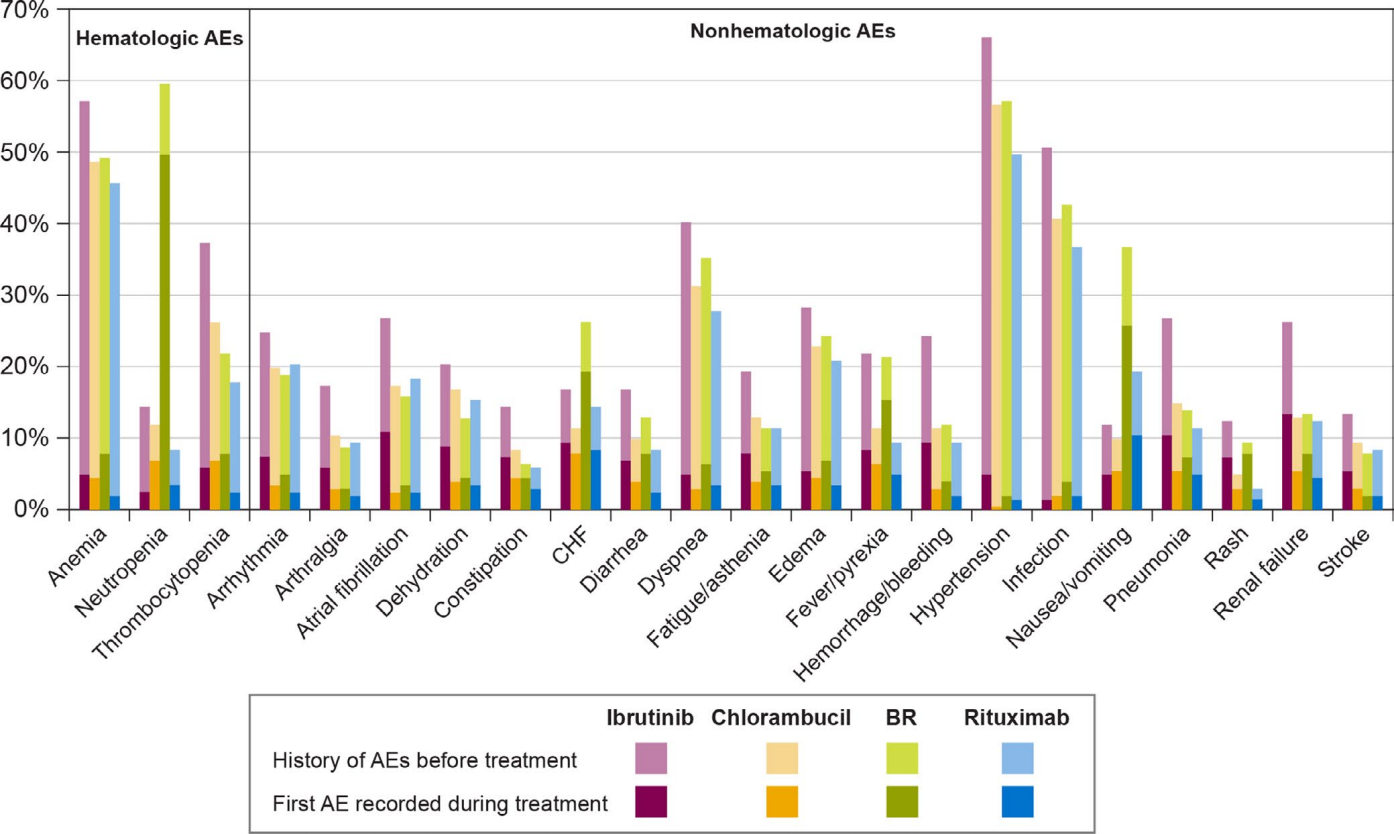
Percentage of patients with adverse events during CLL therapies. AE, adverse event; BR, bendamustine +rituximab; CHF, congestive heart failure; CLL, chronic lymphocytic leukemia. Note: Adverse events occurring in >10% of patients in any treatment group are presented. Within each bar, the darker section represents the proportion of patients who had the event recorded for the first time in their claims history during the specified treatment regimen, whereas the lighter section represents patients who had the event during the specified treatment but also had a history of the event before treatment was initiated

A description of the most frequent AEs during the common CLL regimens across all observed lines of treatment is presented in Figure 1. The AEs are listed alphabetically within the hematologic and the non‐hematologic AEs; only those AEs that appeared in the claims of at least 10% of patients who received any one of the four most common treatments at any time in the follow‐up are presented. Results are presented both as prevalent (regardless of any history of same AE) and incident AEs (recorded for the first time).

Anemia was common with all treatments but was usually recorded for the first time before treatment was started, probably because it is a common manifestation of CLL. Neutropenia was more common in the BR group than the others, as were dehydration and nausea or vomiting. In the ibrutinib group, thrombocytopenia, atrial fibrillation, bleeding, pneumonia, and renal failure were more common than in the other treatment groups. Chlorambucil and rituximab were not prominently associated with particular adverse events in relation to the other treatments.

In an analysis of the number of unique AEs experienced by patients during the follow‐up period, irrespective of the regimen type, 0.3% (*n* = 20) had 0 AEs, 5.2% (*n* = 411) had 1–2 AEs, 20.1% (*n* = 1,597) had 3–5 AEs, and 74.5% (*n* = 5,937) had ≥6 AEs. We also analyzed the number of unique AEs experienced in the first observed line of therapy within the four most common regimens: BR (21.1% had 0–2 AEs, 78.9% had ≥3 AEs); chlorambucil monotherapy (34.8% had 0–2 AEs, 65.2% had ≥3 AEs); rituximab monotherapy (42.5% had 0–2 AEs, 57.5% had ≥3 AEs); and ibrutinib monotherapy (14.4% had 0–2 AEs, 85.6% had ≥3 AEs).

### 3.5 HCRU and costs

**FIGURE 2:**
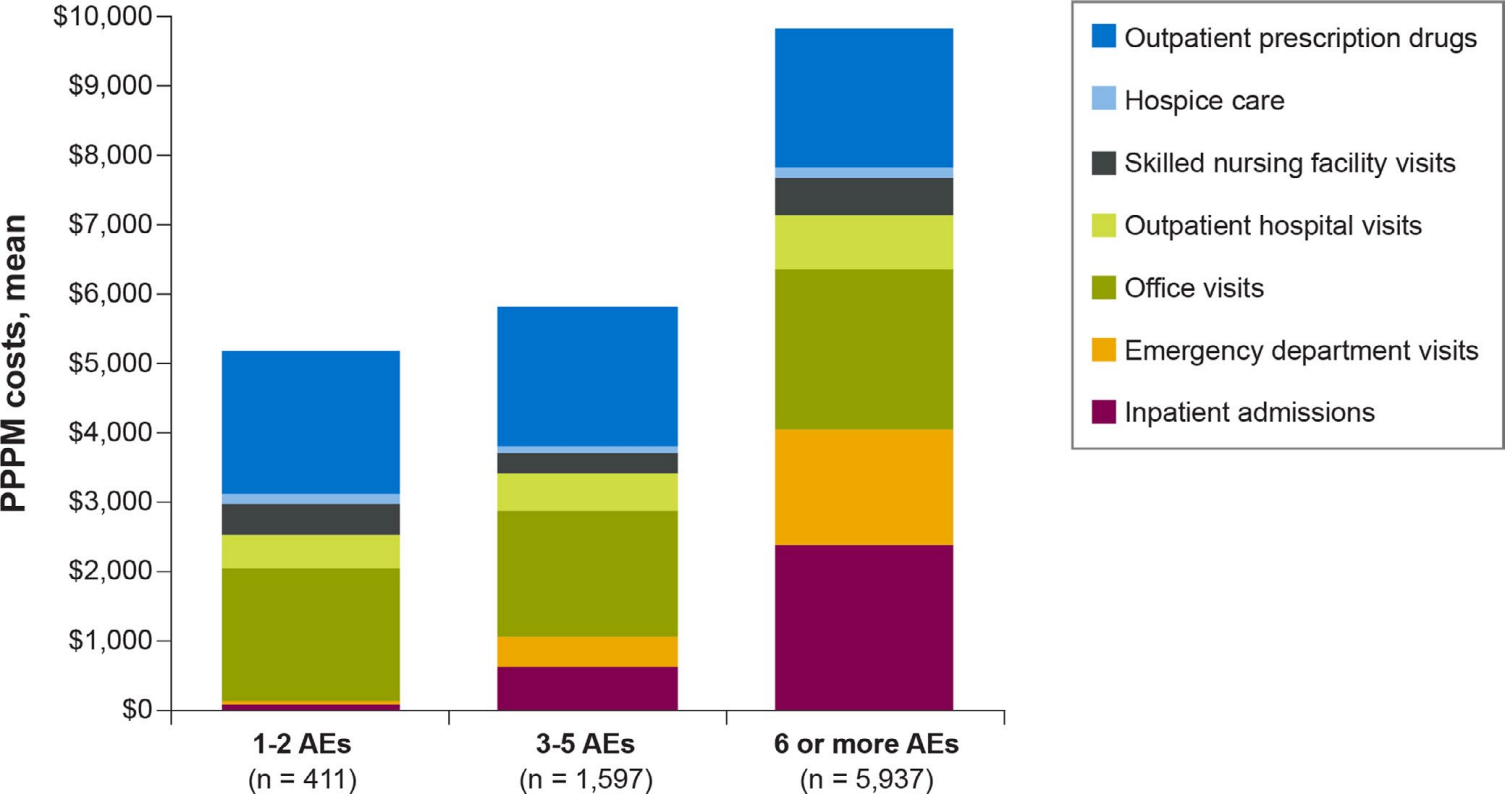
Monthly all‐cause costs by type of service and number of adverse events. AE, adverse event, PPPM, per patient per month. Note: Data for patients with 0 AEs (*n* = 20) are not presented due to very small sample size

Mean PPPM costs were $8,798 (SD = $11,063) for all‐cause and $6,241 (SD = $8,875) for CLL‐related visits and services during the follow‐up period (encompassing all observed lines). The mean PPPM costs for office visit ($2,166 [SD = $2,752]), inpatient admissions ($2,066 [SD = $5,510]), and outpatient prescription drugs ($2,003 [SD = $3,086]) were the largest drivers of the total all‐cause costs. Mean PPPM all‐cause costs during active treatment with ibrutinib, BR, chlorambucil, and R‐monotherapy were $12,192 (SD = $13,788), $4,684 (SD = $8,305), $6,097 (SD = $9,619), and $4,573 (SD = $8,684) respectively. In analyses of costs during active treatment for the observed lines of therapy, the mean PPPM all‐cause costs were $3,340 (SD = $8,148) for the first line, $1,705 (SD = $2,750) for the second line, $496 (SD = $1,033) for the third line, and $788 (SD = $1,544) for the fourth line. Mean PPPM all‐cause costs, measured over the follow‐up period (encompassing all observed lines), increased with the number of unique AEs recorded in the follow‐up period: $5,165 (1–2 AEs; *n* = 411), $5,824 (3–5 AEs; *n* = 1,597), and $9,854 (≥6 AEs; *n* = 5,937) (Figure 2).

For select AEs (anemia, atrial fibrillation, bleeding, infection, and pneumonia), AE‐related PPPM costs during the common treatment regimens were analyzed by care setting; inpatient admissions and emergency department visits were the largest contributors of the AE‐related costs (Table 3).

**TABLE 3:** Per patient per month AE‐related all‐cause costs in the first observed line of therapy

Care Setting	Anemia				Atrial fibrillation				Bleeding				Infection				Pneumonia			
	BR	Clb	Ritux	Ibr	BR	Clb	Ritux	Ibr	BR	Clb	Ritux	Ibr	BR	Clb	Ritux	Ibr	BR	Clb	Ritux	Ibr
Inpatient admissions	$724	$845	$771	$1,319	$335	$317	$344	$710	$108	$131	$106	$357	$632	$791	$698	$1,266	$279	$463	$408	$851
ED visits	$607	$598	$515	$867	$270	$252	$276	$450	$106	$62	$89	$194	$515	$469	$493	$760	$235	$274	$345	$453
Office visits	$527	$85	$885	$66	$39	$23	$41	$45	$30	$10	$17	$26	$125	$63	$95	$99	$31	$26	$32	$55
OP hospital visits	$81	$363	$111	$175	$14	$74	$15	$37	$3	$18	$5	$44	$18	$61	$14	$63	$5	$11	$2	$20
SNF visits	$44	$55	$50	$44	$15	$12	$19	$37	$2	$1	$26	$9	$39	$57	$77	$44	$28	$33	$27	$31
Hospice care	$0	$7	$2	$1	$0	$1	$1	$2	$0	$0	$0	$0	$0	$4	$3	$0	$0	$3	$1	$2
**Total costs**	**$1,984**	**$1,952**	**$2,334**	**$2,473**	**$674**	**$681**	**$696**	**$1,281**	**$249**	**$221**	**$243**	**$631**	**$1,329**	**$1,445**	**$1,379**	**$2,232**	**$578**	**$810**	**$814**	**$1,412**

Abbreviations: BR, bendamustine +rituximab; Clb, chlorambucil; ED, emergency department; Ibr, ibrutinib; OP, outpatient; Ritux, rituximab; SNF, skilled nursing facility.

### 3.6 Factors associated with inpatient admission

Patients who experienced 3 or more AEs were associated with 37 times increased odds of an inpatient admission in the first observed line of therapy than those with 0–2 AEs (odds ratio [OR] = 36.65; 95% CI, 26.32–51.00). Patients aged 85 years or more had 1.5 times higher odds of an inpatient admission versus those aged less than 75 years (OR = 1.52; 95% CI, 1.31–1.78). A CCI score of 3 or more (versus score of 0) was associated with nearly 1.7 times higher odds of an inpatient admission (OR = 1.68; 95% CI, 1.28–2.20). Additionally, female sex; year 2015 for index treatment (vs. 2013); and first observed therapy with BR, R‐monotherapy, and chlorambucil (vs. ibrutinib) were associated with lower odds of an inpatient admission (Table S1).

### 3.7 Factors associated with health care costs

**FIGURE 3:**
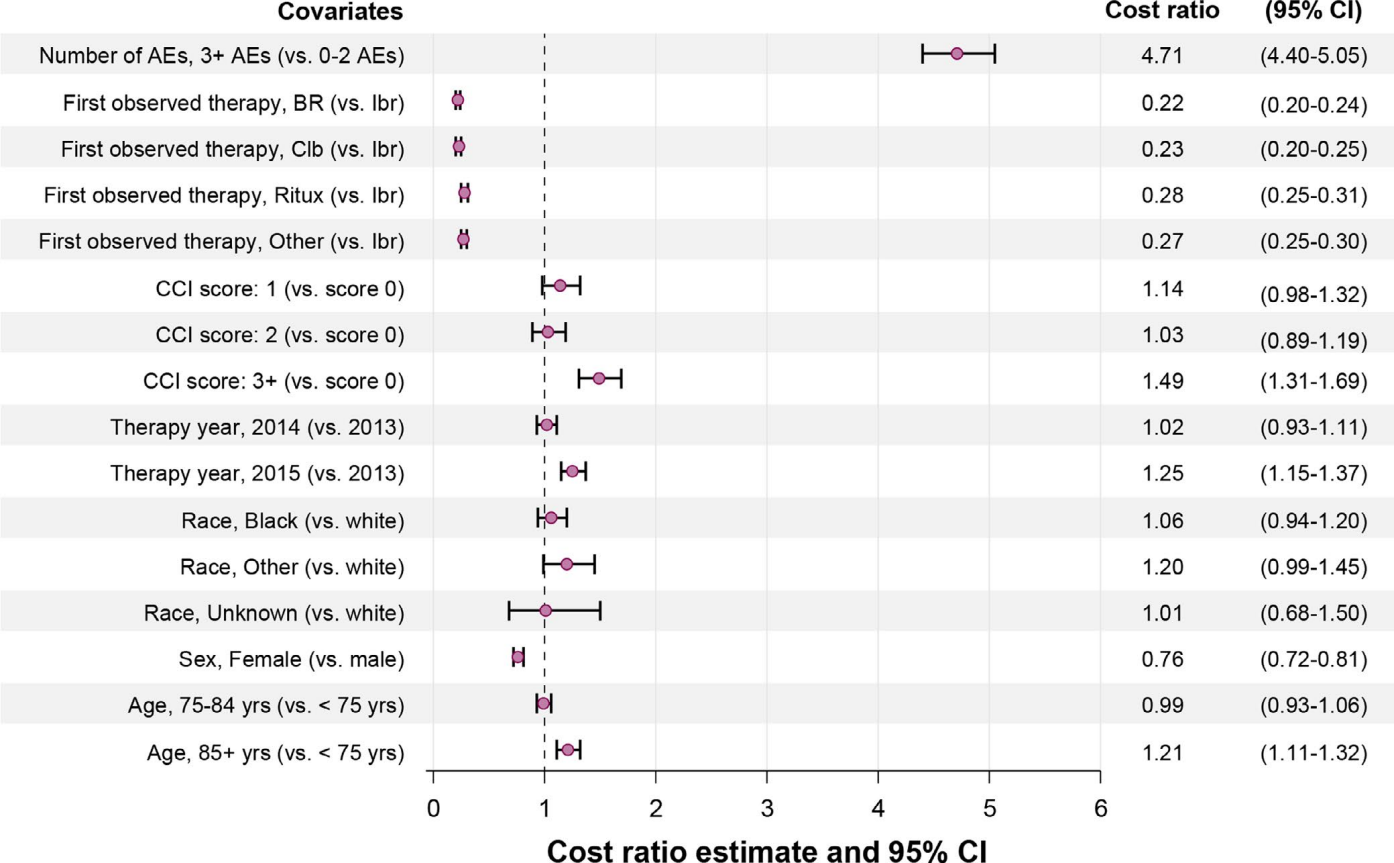
Relative cost estimates from multivariable generalized linear model showing association between baseline patient factors and mean monthly costs during the first observed therapy. AE, adverse event; BR, bendamustine +rituximab; CCI, Charlson Comorbidity Index; CI, confidence interval; Clb, chlorambucil

Patients who experienced 3 or more AEs had 4.7 times higher monthly costs than those with 0–2 AEs during the first observed line of therapy (cost ratio [CR] = 4.71; 95% CI, 4.40–5.05). Patients aged 85 years or more had higher costs than those aged under 75 years (CR = 1.21; 95% CI, 1.11–1.32). Females had higher costs than males. A high CCI score (3 or greater) at baseline predicted higher costs. Patients who received any of the non‐ibrutinib containing regimens in the first observed line had much lower costs than the ibrutinib group. The adjusted CR estimates for all covariates included in the model are displayed in the forest plot (Figure 3).

## 4 DISCUSSION

This study provides a detailed account of treatment patterns and health outcomes, including AEs, OS, and economic burden, in a Medicare population of patients with CLL initiating systemic therapy during 2013–2015. The most common systemic therapy regimens used in the management for CLL were identified in older adults during a period when biologic and targeted therapies were approved and became available in routine clinical practice. The median age, comorbidity burden and high monthly costs during the baseline period suggest that the study is representative of relatively frail population.

The baseline patient characteristics provide evidence of a substantial heterogeneity among the groups of patients initiating the four most common treatments in our study as described in Table 1. The ibrutinib and BR patients were younger on average than those who received chlorambucil or rituximab. The ibrutinib and BR groups also had higher proportions of males than the other two groups. The proportions of patients in the ibrutinib group who entered the study in 2014 or 2015 were larger than in the other groups, chiefly because ibrutinib was first approved for CLL in 2014. As a result of the differences in year of index date, the follow‐up time is shorter for the ibrutinib group than the others. We computed each patients’ atrial fibrillation risk score using a published method^16^ and found that the proportions with a high risk score were higher in the chlorambucil and rituximab groups than in the other groups. The CCI scores tended to be a bit higher for the rituximab patients than the other groups. Lastly, the mean monthly per patient costs during the baseline period were higher for the ibrutinib patients than for those in the other groups. Because the four treatment groups are dissimilar in various ways, any observed differences in outcomes among the groups should not be interpreted as necessarily being causally related to treatment differences.

Ibrutinib was the most frequent regimen among the first observed therapies, followed by chlorambucil. For most of the study period, ibrutinib was approved for relapsed/refractory CLL (first approved in 2014 for relapsed/refractory CLL and in 2016 for frontline) and therefore it is possible that some of the ibrutinib patients in the first observed line may have had received other treatment(s) prior to the 18‐month baseline period imposed in our analysis but were not observable due to absence of longer historical data. It is also likely that the lack of myelosuppressiveness and better tolerability profile of ibrutinib, as compared with chemotherapy‐based regimens, may have made it a suitable alternative for the older, and likely somewhat frail, patient population represented in our analysis. It is notable that among patients who discontinued ibrutinib in the first observed line (excluding those who had a loss of study follow‐up or death), a small proportion of patients (6.3%) reinitiated ibrutinib in the second observed line, suggesting a possible “re‐challenge” or resumption of use after a drug holiday. The overall utilization of other newer targeted therapies approved for CLL at the time of this study, including ofatumumab, obinutuzumab, idelalisib, and venetoclax, appeared to be low (less than 6% across all observed lines). These treatment patterns appear to be largely consistent with the recommended standard of care during the period of this study.

In this study, OS outcomes for each of the four most commonly observed regimens were analyzed separately because of (1) potential differences among patients that may drive treatment decisions and, as a result, affect survival outcomes and (2) potential misclassification of line of therapy. Reported OS results from clinical trials should also not be compared directly with those in our study, but they do provide some context. Twenty‐four‐month OS in recently published data from a clinical trial was 95% for BR and 90% for ibrutinib in the first‐line setting.^23^ In the relapsed/refractory setting, 24‐month OS in other clinical trials was approximately 80% for BR^24^ and nearly 83% for ibrutinib.^25^ For chlorambucil in the first‐line setting, 24‐month OS was reported to be nearly 85%.^26^ In our study, the 24‐month survival rates ranged between 68% and 79% depending upon the type of treatment, suggesting that the survival rates in Medicare patients treated with the commonly observed regimens in the real‐world setting may be somewhat lower than those seen in clinical trials. This is not surprising in view of the multiple selection factors for participating in clinical trials and some uncertainty about actual line of therapy in the present study. The exception to this is what we observed for rituximab. For rituximab monotherapy in the relapsed setting, the 24‐month OS reported in a clinical trial was 53%^27^—a rate lower than that observed in our study. However, survival estimates in a relapsed disease setting are expected to be lower than in the first‐line setting, and although we cannot be certain that all first observed therapies in our analysis are truly first‐line therapies, this difference is not surprising. The median OS estimate was not obtained for all regimens, except chlorambucil, because of the long survival times and relatively short study follow‐up, resulting in high rates of censoring.

The AE rates observed in our study are representative of a population of patients treated in routine clinical settings. Therefore, these rates may differ from those observed in randomized clinical trials, which tend to include relatively younger and healthier patients, and in which the reporting of adverse events is done prospectively based on clinical assessments rather than retrospectively from claims diagnoses. The association of particular AEs with a given treatment may represent treatment effects, but some results may reflect patient selection for particular treatments. For example, patients with renal insufficiency may have been treated with ibrutinib preferentially because its metabolites are excreted in feces,^28^ whereas bendamustine is largely excreted in urine and should not be used in patients with creatinine clearance <30 mL/min.^29^ Therefore, no causal association between the AEs and treatments should be drawn in the absence of randomization and given the evidence of imbalance between the treatment groups on baseline patient characteristics that were previously discussed.

The results of this study indicate that resource utilization and the economic burden associated with CLL are substantial. We observed a large increase in the economic burden from the baseline period to the period after CLL therapy initiation. Health care costs during the common CLL therapies observed among Medicare patients in this study are lower than that reported among patients with CLL enrolled in private health plans.^8^, ^9^ This finding is consistent with previous studies highlighting that Medicare payment rates are lower than those of private health insurers.^30^, ^31^ Extrapolating from the mean monthly cost of $8,798, as observed in the postindex period in this study, the projected annual economic burden to Medicare for each patient with CLL initiating systemic therapy would be approximately $106,000. Furthermore, with the projected increase in the prevalence of CLL,^7^ the financial burden to Medicare over patient lifetimes could only be expected to increase even further. Our study also showed that the utilization and costs were positively correlated with the number of AEs experienced in the postindex period. The increasing costs with greater number of AEs appeared to be driven primarily by costs associated with inpatient admissions, office visits, and outpatient prescription drugs; in the group experiencing six or more AEs, the emergency department visits also contributed substantially to the overall costs. In assessing costs for select AEs, our analysis showed that the AE‐related costs predominantly comprised inpatient admissions and emergency department visits. These results, stratified by treatment regimen, also suggest that the costs for utilization of services associated with the AEs were consistently higher for ibrutinib (the only BTK inhibitor available during the study period) as compared with chemotherapy‐based regimens (BR and chlorambucil) or rituximab monotherapy. In the multivariable analyses, patients who experienced three or more AEs were found to be associated with nearly 37 times higher odds of an inpatient admission and nearly five times higher monthly costs than those with 0–2 AEs during the first observed line of therapy. The relationship between AEs and inpatient admissions observed in this study are only correlative (not causal) in nature. Nevertheless, these findings suggest the need to explore and better understand AE consequences and develop therapeutic alternatives that potentially induce fewer toxicities and that may offer improved clinical outcomes at lower costs. The multivariable analysis also showed that patients treated with BR, rituximab, and chlorambucil had lower odds of inpatient admission as well as lower monthly costs as compared with those treated with ibrutinib, suggesting a potentially higher disease burden in Medicare patients initiating treatment with ibrutinib. However, this could not be directly confirmed in our study because no information was available on disease severity, patients’ functional status, and long‐term treatment history (i.e., in the period prior to the 18‐month baseline).

In interpreting the study findings, certain limitations that are generally present in studies based on retrospective claims databases should be considered. The selection of the study cohort was based on diagnosis codes indicative of CLL as recorded in insurance claims, and any erroneous coding could have misclassified patients. In the absence of access to patients’ medical records, this study assumed that claims associated with treatments, AEs, and costs were accurately coded. Moreover, data on clinical stage, performance status, and prognostic factors (e.g., 17p deletion, TP53 mutation), which could have influenced treatment selection, AEs, and costs, were not available and accounted for in our analysis. Because all payments associated with claims containing a diagnosis and/or procedure code for the AE in question were attributed to the AE, there may be overlaps and possible overestimation of individual AE‐related costs. Conversely, costs not coded as relating to an AE but still spent toward managing the AE were unaccounted for, which may have offset some of the overestimation. Although an 18‐month baseline period was required to identify patients receiving initial treatment for CLL, it is likely that many patients with more than 18 months of treatment‐free time related to previously diagnosed CLL were included, and therefore, the first observed therapy may not have been the true first‐line treatment for a substantial proportion of patients and likewise for all subsequently observed lines of therapy; this is especially true for patients treated with ibrutinib, which was approved for use in a relapsed/refractory setting during most of the study period. The study also lacked clinical details necessary to verify the cause of an AE occurrence; therefore, no causal association between treatments and AEs can be established. Additionally, claims data do not contain other information on AEs, including severity, duration, and actions taken. For a small fraction of patients who discontinued and later reinitiated treatment with ibrutinib, our data lacked information on reason for discontinuation (intolerance or progression) to assess the status of patients who reinitiated ibrutinib. The time period of data availability for Medicare has a significant lag; so, although the data used in this study were the most recent available at the time the study was initiated, the results may not reflect CLL treatment patterns today. Several therapeutic alternatives became available, and existing therapies received a new indication over the period of our study; therefore, the uptake of these therapies is not adequately represented. For instance, ibrutinib was first approved in February 2014 for patients with CLL who have received at least one previous therapy, but it later received approval for first‐line treatment in March 2016 (toward the end of our study period); ofatumumab, another anti‐CD20 monoclonal antibody, was approved in April 2014 for previously untreated patients for whom fludarabine‐based therapy was considered inappropriate; idelalisib, a kinase inhibitor, was approved in July 2014 for patients with relapsed CLL; venetoclax, a BCL‐2 inhibitor, was approved in April 2016 for previously treated patients with CLL with 17p deletion. Furthermore, some more recently approved therapies are not represented at all in this study (e.g., duvelisib, a newer phosphoinositide 3‐kinase inhibitor [approved September 2018], and acalabrutinib, a novel Bruton's tyrosine kinase inhibitor [approved November 2019], both approved for relapsed/refractory CLL). It is important that the effects of such newer therapies on practice patterns, survival, AE rates, and health care costs are assessed in future research.

## 5 CONCLUSIONS

To our knowledge, this is the largest contemporary observational study reporting outcomes among patients with CLL initiating treatments in a real‐world setting. Over two‐thirds of patients survived at least 2 years after the start of their first observed therapy during the study period. The study findings highlight considerable susceptibility to AEs and an unmet medical need in Medicare patients with CLL treated in routine practice. Medicare incurs substantial economic burden following initiation of systemic therapy for CLL, and greater numbers of AEs were associated with higher overall cost of CLL management.

## CONFLICT OF INTEREST

RKG, SPN, KLD, and JAK are full‐time employees of RTI Health Solutions, which received funding from AstraZeneca to conduct this research. RTI Health Solutions is a business unit of Research Triangle Institute, which is an independent, nonprofit, research organization that does work for government agencies and private companies. SMK was full‐time employee of AstraZeneca, the funding organization for this analysis, during study conduct and manuscript development, and HL is full‐time employee of AstraZeneca. Authors have no other conflict of interest to declare.

## ETHICAL STATEMENT

This study was reviewed by the Institutional Review Board at RTI International and received the “not human research” determination.

## Supporting information

Supplementary Material

## Data Availability

The data that support the findings of this study are available from the Centers for the Medicare and Medicaid Services through the Research and Data Assistance Center. Restrictions apply to the availability of these data, which were used under license for this study.

## References

[1] American Cancer Society . Cancer Facts & Figures. 2020. Available at: https://www.cancer.org/content/dam/cancer‐org/research/cancer‐facts‐and‐statistics/annual‐cancer‐facts‐and‐figures/2020/cancer‐facts‐and‐figures‐2020.pdf. Accessed: June 9, 2020.

[2] Nabhan C , Rosen ST . Chronic lymphocytic leukemia: a clinical review. JAMA. 2014;312(21):2265‐2276. 25461996 10.1001/jama.2014.14553

[3] National Comprehensive Cancer Network . Clinical practice guidelines in oncology (NCCN Guidelines®): chronic lymphocytic leukaemia/small lymphocytic lymphoma. 2020. Available at: https://www.nccn.org/professionals/physician_gls/default.aspx. Accessed: May 26, 2020.

[4] American Cancer Society . Cancer Facts & Figures: 2018. Available at: https://www.cancer.org/research/cancer‐facts‐statistics/all‐cancer‐facts‐figures/cancer‐facts‐figures‐2018.html. Accessed: May 4, 2020.

[5] Hao T , Li‐Talley M , Buck A , Chen W . An emerging trend of rapid increase of leukemia but not all cancers in the aging population in the United States. Sci Rep. 2019;9(1):12070. 31427635 10.1038/s41598-019-48445-1 PMC6700310

[6] American Cancer Society . About chronic lymphocytic leukemia: Key statistics for chronic lymphocytic leukemia. Jan 8, 2020. Available at: https://www.cancer.org/cancer/chronic‐lymphocytic‐leukemia/about/key‐statistics.html. Accessed: May 9, 2020.

[7] Chen Q , Jain N , Ayer T , et al. Economic burden of chronic lymphocytic leukemia in the era of oral targeted therapies in the United States. J Clin Oncol. 2017;35(2):166‐174. 27870563 10.1200/JCO.2016.68.2856 PMC5559889

[8] Kabadi SM , Goyal RK , Nagar SP , Kaye JA , Davis KL . Treatment patterns, adverse events, and economic burden in a privately insured population of patients with chronic lymphocytic leukemia in the United States. Cancer Med. 2019;8(8):3803‐3810. 31144473 10.1002/cam4.2268 PMC6639180

[9] Reyes C , Gauthier G , Shi S , Guerin A . Overall survival and health care costs of Medicare patients with previously treated chronic lymphocytic leukemia. J Cancer Ther. 2018;9(07):576.

[10] Mato A , Jahnke J , Li P , et al. Real‐world treatment and outcomes among older adults with chronic lymphocytic leukemia before the novel agents era. Haematologica. 2018;103(10):e462‐e465. 29700170 10.3324/haematol.2017.185868 PMC6165790

[11] Satram‐Hoang S , Reyes C , Hoang KQ , Momin F , Skettino S . Treatment practice in the elderly patient with chronic lymphocytic leukemia—analysis of the combined SEER and Medicare database. Ann Hematol. 2014;93(8):1335‐1344. 24638841 10.1007/s00277-014-2048-6 PMC4082137

[12] Hess GP , Chen C , Satram‐Hoang S , Reyes CM . Characteristics and treatment patterns in patients newly diagnosed with chronic lymphocytic leukemia (CLL). J Clin Oncol. 2010;28(15 suppl).e16561

[13] Seymour EK , Ruterbusch JJ , Beebe‐Dimmer JL , Schiffer CA . Real‐world testing and treatment patterns in chronic lymphocytic leukemia: A SEER patterns of care analysis. Cancer. 2019;125(1):135‐143. 30343488 10.1002/cncr.31738 PMC6309467

[14] Olszewski AJ , Davids MS , Yakirevich I , Egan PC . Early adoption and outcomes of ibrutinib as treatment for older patients with chronic lymphocytic leukemia (CLL): a population‐based study. Blood. 2019;134(Suppl 1):265.

[15] Charlson ME , Charlson RE , Peterson JC , Marinopoulos SS , Briggs WM , Hollenberg JP . The Charlson comorbidity index is adapted to predict costs of chronic disease in primary care patients. J Clin Epidemiol. 2008;61(12):1234‐1240. 18619805 10.1016/j.jclinepi.2008.01.006

[16] Chyou JY , Hunter TD , Mollenkopf SA , Turakhia MP , Reynolds MR . Individual and combined risk factors for incident atrial fibrillation and incident stroke: an analysis of 3 million at‐risk US patients. J Am Heart Assoc. 2015;4(7).e001723 10.1161/JAHA.114.001723 PMC4608064 26206736

[17] National Comprehensive Cancer Network . Clinical practice guidelines in oncology (NCCN Guidelines®): chronic lymphocytic leukaemia/small lymphocytic lymphoma. 2015;Version 2.2015.

[18] National Comprehensive Cancer Network . Clinical practice guidelines in oncology (NCCN Guidelines®): chronic lymphocytic leukaemia/small lymphocytic lymphoma. 2017;Version 1.2017.

[19] Goyal RK , Nagar SP , Kabadi SM , Kaye JA , Seal B , Mato AR . Adverse events, resource use, and economic burden associated with mantle cell lymphoma: a real‐world assessment of privately insured patients in the United States. Leuk Lymphoma. 2019;60(4):955‐963. 30277099 10.1080/10428194.2018.1509320 PMC7564890

[20] Danese MD , Reyes CM , Gleeson ML , Halperin M , Skettino SL , Mikhael J . Estimating the population benefits and costs of rituximab therapy in the United States from 1998 to 2013 using real‐world data. Med Care. 2016;54(4):343. 26759977 10.1097/MLR.0000000000000486 PMC4795096

[21] Hurvitz S , Guerin A , Brammer M , et al. Investigation of adverse‐event‐related costs for patients with metastatic breast cancer in a real‐world setting. Oncologist. 2014;19(9):901‐908. 25085897 10.1634/theoncologist.2014-0059 PMC4153460

[22] Griffiths RI , Gleeson ML , Mikhael J , Dreyling MH , Danese MD . Comparative effectiveness and cost of adding rituximab to first‐line chemotherapy for elderly patients diagnosed with diffuse large B‐cell lymphoma. Cancer. 2012;118(24):6079‐6088. 22648454 10.1002/cncr.27638

[23] Woyach JA , Ruppert AS , Heerema NA , et al. Ibrutinib regimens versus chemoimmunotherapy in older patients with untreated CLL. N Engl J Med. 2018;379(26):2517‐2528. 30501481 10.1056/NEJMoa1812836 PMC6325637

[24] Fraser G , Cramer P , Demirkan F , et al. Updated results from the phase 3 HELIOS study of ibrutinib, bendamustine, and rituximab in relapsed chronic lymphocytic leukemia/small lymphocytic lymphoma. Leukemia. 2019;33(4):969‐980. 30315239 10.1038/s41375-018-0276-9 PMC6484712

[25] Munir T , Brown JR , O'Brien S , et al. Final analysis from RESONATE: Up to six years of follow‐up on ibrutinib in patients with previously treated chronic lymphocytic leukemia or small lymphocytic lymphoma. Am J Hematol. 2019;94(12):1353‐1363. 31512258 10.1002/ajh.25638 PMC6899718

[26] Burger JA , Tedeschi A , Barr PM , et al. Ibrutinib as initial therapy for patients with chronic lymphocytic leukemia. N Engl J Med. 2015;373(25):2425‐2437. 26639149 10.1056/NEJMoa1509388 PMC4722809

[27] Sharman JP , Coutre SE , Furman RR , et al. Final results of a randomized, phase III study of rituximab with or without idelalisib followed by open‐label idelalisib in patients with relapsed chronic lymphocytic leukemia. J Clin Oncol. 2019;37(16):1391‐1402. 30995176 10.1200/JCO.18.01460 PMC10448866

[28] Imbruvica (ibrutinib) FDA Prescribing Information . November 2019. Available at: https://www.accessdata.fda.gov/drugsatfda_docs/label/2019/205552s029,210563s004lbl.pdf Accessed: June 17, 2020 .

[29] Treanda (bendamustine hydrochloride) FDA Prescribing Information . November 2019. Available at: https://www.accessdata.fda.gov/drugsatfda_docs/label/2019/022249s024lbl.pdf.

[30] Clemens J , Gottlieb JD . In the shadow of a giant: Medicare’s influence on private physician payments. J Polit Econ. 2017;125(1):1‐39. 28713176 10.1086/689772 PMC5509075

[31] Wallace J , Song Z . Traditional Medicare versus private insurance: how spending, volume, and price change at age sixty‐five. Health Aff. 2016;35(5):864‐872. 10.1377/hlthaff.2015.1195 PMC4943661 27140993

