# Supplementary material for: Overall survival, adverse events, and economic burden in patients with chronic lymphocytic leukemia receiving systemic therapy: Real‐world evidence from the medicare population
Source: Cancer Med. 2021 Mar 18;10(8):2690–702. doi: 10.1002/cam4.3855 (PMC8026937; doi:10.1002/cam4.3855)
Supplement: Supplementary file 1 — Supplementary Material [file CAM4-10-2690-s001.docx]

**Supplemental Figure 1. Study Design Schematic**

**
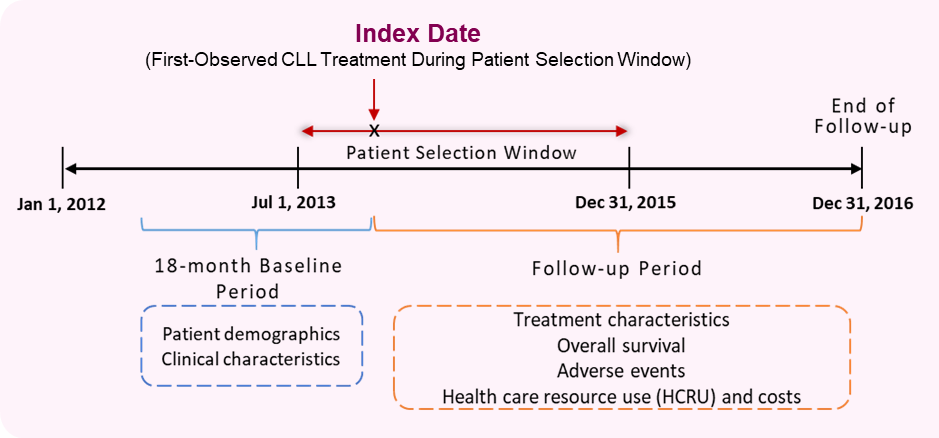
**

**Supplemental Figure 2. Sample Attrition Flowchart for Patients With Chronic Lymphocytic Leukemia**

Supplemental Figure 3. Overall Survival From Start of First Observed Treatment Through Death or the End of Study Follow-up


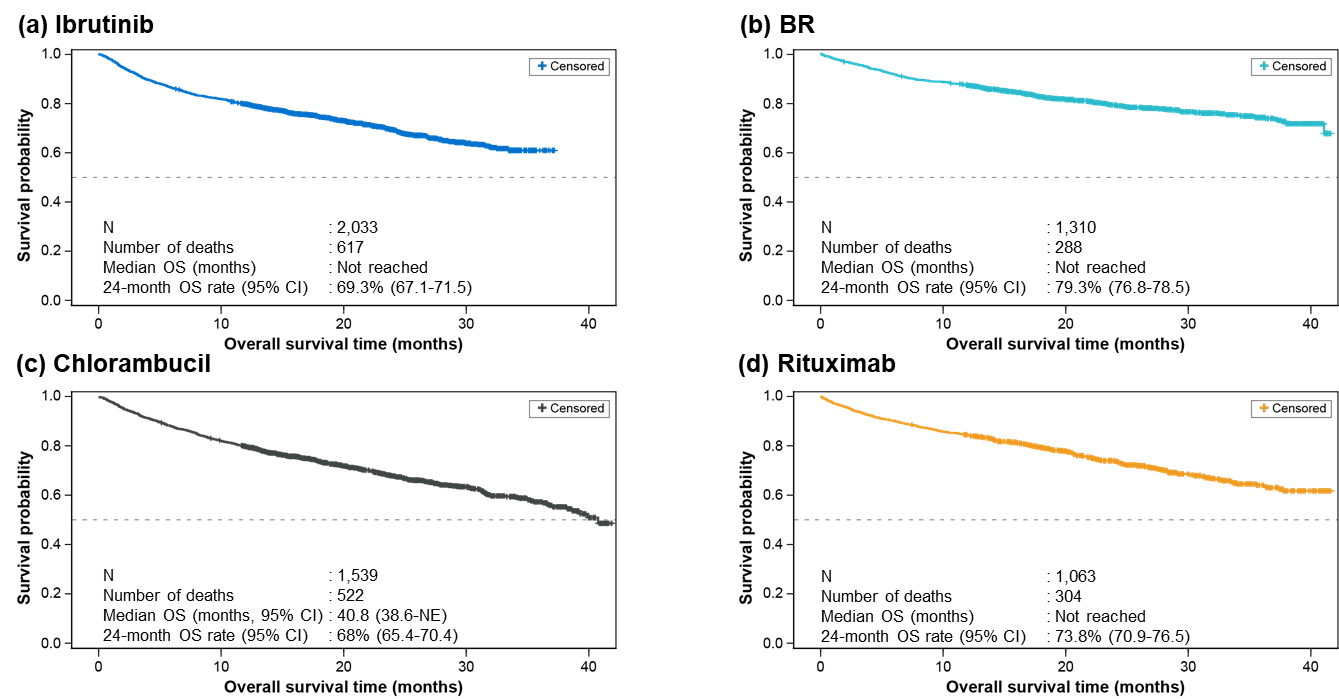


BR = bendamustine + rituximab; CI = confidence interval; NE = not estimable; OS = overall survival.

**Supplemental Table 1. Multivariable Logistic Regression Model Assessing Association Between Number of Adverse Events and Inpatient Admission During the First Observed Therapy Line (N = 7,965)**

| **Covariates** | **Odds Ratio** | **95% CI** | |
| --- | --- | --- | --- |
| **Number of Adverse Events** (ref = 0 to 2 AEs) |  |  |  |
| ≥ 3 AEs | **36.64** | **26.32** | **51.00** |
| **Age at Index** (ref = ≤ 74 years) |  |  |  |
| 75 - 84 years | 1.09 | 0.97 | 1.23 |
| ≥ 85 years | **1.52** | **1.31** | **1.78** |
| **Sex** (ref = male) |  |  |  |
| Female | **0.82** | **0.74** | **0.92** |
| **Year of Index Treatment** (ref = 2013) |  |  |  |
| 2014 | 0.99 | 0.84 | 1.17 |
| 2015 | **0.82** | **0.69** | **0.96** |
| **Race (ref = White)** |  |  |  |
| Black | 1.03 | 0.83 | 1.27 |
| Others | 1.00 | 0.71 | 1.42 |
| Unknown | 0.79 | 0.38 | 1.64 |
| **CCI Score (ref = 0)** |  |  |  |
| 1 | 1.17 | 0.85 | 1.61 |
| 2 | 1.11 | 0.82 | 1.52 |
| 3+ | **1.68** | **1.28** | **2.20** |
| **First Observed Treatment Regimen** (ref = Ibrutinib monotherapy) |  |  |  |
| BR | **0.51** | **0.43** | **0.60** |
| Rituximab monotherapy | **0.41** | **0.34** | **0.51** |
| Chlorambucil | **0.46** | **0.39** | **0.55** |
| Other regimens | **0.63** | **0.54** | **0.73** |

AEs = adverse events; BR = bendamustine/rituximab; CCI = Charlson comorbidity index; CI = confidence interval; ref = reference.
